# Brivaracetam and Levetiracetam Suppress Astroglial L-Glutamate Release through Hemichannel via Inhibition of Synaptic Vesicle Protein

**DOI:** 10.3390/ijms23094473

**Published:** 2022-04-19

**Authors:** Kouji Fukuyama, Motohiro Okada

**Affiliations:** Department of Neuropsychiatry, Division of Neuroscience, Graduate School of Medicine, Mie University, Tsu 514-8507, Japan; k-fukuyama@clin.medic.mie-u.ac.jp

**Keywords:** astrocyte, brivaracetam, levetiracetam, epilepsy, synaptic vesicle protein 2A, connexin43

## Abstract

To explore the pathophysiological mechanisms of antiseizure and adverse behavioural/psychiatric effects of brivaracetam and levetiracetam, in the present study, we determined the effects of brivaracetam and levetiracetam on astroglial L-glutamate release induced by artificial high-frequency oscillation (HFO) bursts using ultra-high-performance liquid chromatography. Additionally, the effects of brivaracetam and levetiracetam on protein expressions of connexin43 (Cx43) and synaptic vesicle protein 2A (SV2A) in the plasma membrane of primary cultured rat astrocytes were determined using a capillary immunoblotting system. Acutely artificial fast-ripple HFO (500 Hz) burst stimulation use-dependently increased L-glutamate release through Cx43-containing hemichannels without affecting the expression of Cx43 or SV2A in the plasma membrane, whereas acute physiological ripple HFO (200 Hz) stimulation did not affect astroglial L-glutamate release or expression of Cx43 or SV2A. Contrarily, subchronic ripple HFO and acute pathological fast-ripple HFO (500 Hz) stimulations use-dependently increased L-glutamate release through Cx43-containing hemichannels and Cx43 expression in the plasma membrane. Subchronic fast-ripple HFO-evoked stimulation produced ectopic expression of SV2A in the plasma membrane, but subchronic ripple HFO stimulation did not generate ectopic SV2A. Subchronic administration of brivaracetam and levetiracetam concentration-dependently suppressed fast-ripple HFO-induced astroglial L-glutamate release and expression of Cx43 and SV2A in the plasma membrane. In contrast, subchronic ripple HFO-evoked stimulation induced astroglial L-glutamate release, and Cx43 expression in the plasma membrane was inhibited by subchronic levetiracetam administration, but was not affected by brivaracetam. These results suggest that brivaracetam and levetiracetam inhibit epileptogenic fast-ripple HFO-induced activated astroglial transmission associated with hemichannels. In contrast, the inhibitory effect of therapeutic-relevant concentrations of levetiracetam on physiological ripple HFO-induced astroglial responses probably contributes to the adverse behavioural/psychiatric effects of levetiracetam.

## 1. Introduction

Modulation of the function of synaptic vesicle protein type 2A (SV2A) has been considered to play an important role in the suppression of epileptic seizure, since high-affinity SV2A-binding compounds, levetiracetam and brivaracetam, are first-line antiseizure drugs [1,2]. Indeed, some meta-analysis studies reported that levetiracetam and brivaracetam displayed the best efficacy and safety profiles in the class of antiseizure drugs [3,4,5]. In spite of the effectiveness of levetiracetam and brivaracetam, these two agents have been denoted as high-risk antiseizure drugs of aggressive behaviour [6,7,8,9,10]. Considering the impacts on quality of life, adverse behavioural or psychiatric events induced by antiseizure drugs play important roles in the decisions regarding antiepileptic medication [6,11]. However, the detailed mechanisms of aggressive behaviour induced by levetiracetam and brivaracetam have yet to be clarified.

SV2A is ubiquitously expressed in various structures in the central nervous system but selectively expressed in the synaptic vesicle membrane, with five copies in each vesicle [12,13]. It has been established that SV2A contributes to Ca^2+^-sensitive exocytosis of transmitters via Ca^2+^-dependent interaction with synaptotagmin, vesicular transporting, stabilisation of vesicular loading of neurotransmitter, vesicular proteins anchoring, assistance in vesicle trafficking, and regulation of Ca^2+^ sensitivity [13,14,15]. Additionally, ectopic SV2A in the plasma membrane induced by hyper-neuronal activations probably generates the matrix with laminin type 1 receptor in the extracellular space or connexin43 (Cx43) in the plasma membrane [13,16,17]. The connexin family contributes to the regulation of transmission and homeostasis in the central nervous system [18,19]. The six assembling connexins (connexon) in the plasma membrane connect with the other connexon in the plasma membrane in various neighbouring cells, neurons, astrocytes, oligodendrocytes, and microglia, forming a gap junction [20]. The gap junction is involved in physiological functions, such as neuronal excitability, synaptic plasticity, tripartite synaptic transmission, and homeostasis maintenance in the central nervous system [18,19,20,21,22]. Contrarily, a single connexon is one of the responsible molecules in tripartite synaptic transmission (among the presynaptic and postsynaptic neurones as well as their intimate association with surrounding glia cells) [19,21,22] as a hemichannel, which also regulates ionic homeostasis, including ionic movement regulation among intracellular/extracellular spaces and the release of several gliotransmitters—including D-serine, L-glutamate, prostaglandins, adenosine triphosphate (ATP), and nicotinamide adenine dinucleotide—which are involved in autocrine/paracrine signalling [18,23,24,25].

Astroglial hemichannels are low opening probabilities during the resting stage; however, the hyperactivations—such as hyper-depolarisation, extracellular/intracellular imbalance of cations (elevations in extracellular K^+^ and reductions in extracellular Ca^2+^), and pH activate hemichannels—result in the release of astroglial transmitters [26]. Interestingly, a recent in vivo study revealed that astroglial L-glutamate release induced by high-frequency oscillation (HFO) bursts contribute to the pathophysiology of the carbamazepine-resistant and zonisamide-sensitive autosomal-dominant sleep-related hypermotor epilepsy (ADSHE) [26]. HFO is categorised by two frequency ranges: relatively slow physiological ripple HFO (80–250 Hz and tens of milliseconds duration), which is observed during sleep spindle; and epileptogenic fast-ripple HFO (250–500 Hz and milliseconds duration), which is detected prior to the initiation of epileptic seizure in the epileptogenic zone using wideband electroencephalography [27,28]. It is speculated that sleep spindle/ripple HFO bursts provide the formation and/or maintenance of a part of cognitive function [29]. These previous preclinical and clinical findings suggest that antiseizure drugs that selectively inhibit fast-ripple HFO and do not affect ripple HFO are considered to possess ideal pharmacological properties.

Preclinical studies reported that levetiracetam suppressed the frequency of fast-ripple HFO, whereas the inhibitory effects of levetiracetam on the ripple HFO were weaker compared to fast-ripple HFO [30,31]. A recent systematic review reported that switching from levetiracetam to brivaracetam could improve adverse behavioural/psychiatric reactions induced by levetiracetam [32]. Furthermore, the effects of brivaracetam and levetiracetam on the functional changes of tripartite synaptic transmission induced by HFOs also remain to be clarified. Against this background, to clarify the mechanisms of antiseizure drugs and the adverse behavioural/psychiatric effects of brivaracetam and levetiracetam, we analysed the effects of brivaracetam and levetiracetam on the astroglial L-glutamate release associated with Cx43-containing hemichannel induced by artificial HFOs bursts using ultra-high pressure liquid chromatography. Additionally, we determined the effects of these two antiseizure drugs on the expression of SV2A and Cx43 in the plasma membrane fraction of primary cultured astrocytes using a capillary immunoblotting system.

## 2. Results

The detailed study designs of studies 1–5 are described in Section 4.3 (artificial HFO-evoked stimulation and study designs). According to our previous studies, subchronic administration of target agent and HFO-evoked stimulations were performed during after 21 days of culture (DIV21) and DIV28 (for 7 days) [13,23,26,33]. Acute administration of target agent and HFO-evoked stimulations were performed at DIV28 for 120 min [13,26,34]. In the present study, the rat primary cultured astrocytes were electrically stimulated by two types of artificial HFO burst-like stimulations (HFO-evoked stimulation), ripple HFO and fast-ripple HFO-evoked stimulations, using a busdrive amplifier (SEG-3104MG, Miyuki Giken, Tokyo, Japan) [26]. Sets of ripple HFO and fast-ripple HFO-evoked stimulations were composed of 10 stimuli at 200 Hz and 10 bursts (50% duty cycle) at burst intervals of 100 ms per 1 sec, and 10 stimuli at 500 Hz and 10 bursts (50% duty cycle) at burst intervals of 40 ms per 1 sec, respectively [26]. The energisation amounts during the ripple-evoked and fast-ripple-evoked stimulations were set to be equal [26].

### 2.1. Effects of Acute Artificial HFO-Evoked Stimulations on Astroglial Transmission-Associated Cx43-Containing Hemichannel

#### 2.1.1. Acute Effects of Artificial HFO-Evoked Stimulation on Astroglial L-Glutamate Release (Study 1)

**Figure 1:**
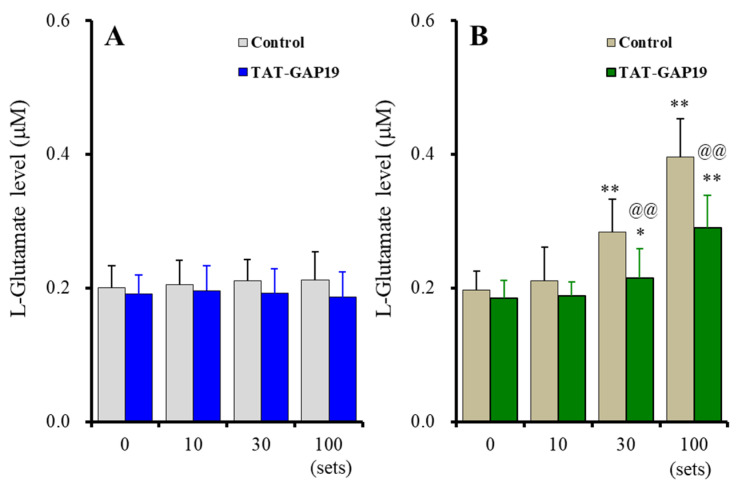
Effects of acute artificial ripple high-frequency oscillation (HFO) (**A**) and fast-ripple-HFO evoked stimulations (**B**) on L-glutamate release from cultured astrocytes (gray and blown columns), and the effects of selective connexin43 (Cx43) inhibitor, TAT-GAP19 (20 μM), on HFOs-evoked astroglial L-glutamate release (blue and green columns). Ordinates indicate the mean ± standard deviation (SD) of extracellular L-glutamate level (μM) (*n* = 6). Abscissas indicate the numbers of HFO-evoked stimulations sets. * *p* < 0.05, ** *p* < 0.01; relative to the HFO-evoked stimulation free (0) and @@ *p* < 0.01; relative to control (TAT-GAP19 free) by multivariate analysis of variance (MANOVA) with Tukey’s post-hoc test.

Acute ripple HFO-evoked stimulation did not affect astroglial L-glutamate release, and the selective Cx43 inhibitor, 20 μM TAT-GAP19, did not affect astroglial L-glutamate release [F_stimulation_(1.7, 17.2) = 0.59 (*p* > 0.1), F_TAT-GAP19_(1, 10) = 0.62 (*p* > 0.1), F_stimulation*TAT-GAP19_ (1.7, 17.2) = 1.46 (*p* > 0.1)] (Figure 1A). The fast-ripple HFO-evoked stimulation use-dependently increased astroglial L-glutamate release, and 20 μM TAT-GAP19 suppressed the fast-ripple HFO-evoked astroglial L-glutamate release [F_stimulation_(3, 30) = 200.46 (*p* < 0.01), F_TAT-GAP19_(1, 10) = 5.27 (*p* < 0.05), F_stimulation*TAT-GAP19_(3, 30) = 19.35 (*p* < 0.01)] (Figure 1B). Ten sets of the fast-ripple HFO-evoked stimulation did not affect L-glutamate release, whereas 30 and 100 sets increased L-glutamate release through astroglial Cx43-containing hemichannels (Figure 1B).

#### 2.1.2. Acute Effects of Artificial HFO-Evoked Stimulation on Expression of Cx43 Protein in the Plasma Membrane (Study 1)

**Figure 2:**
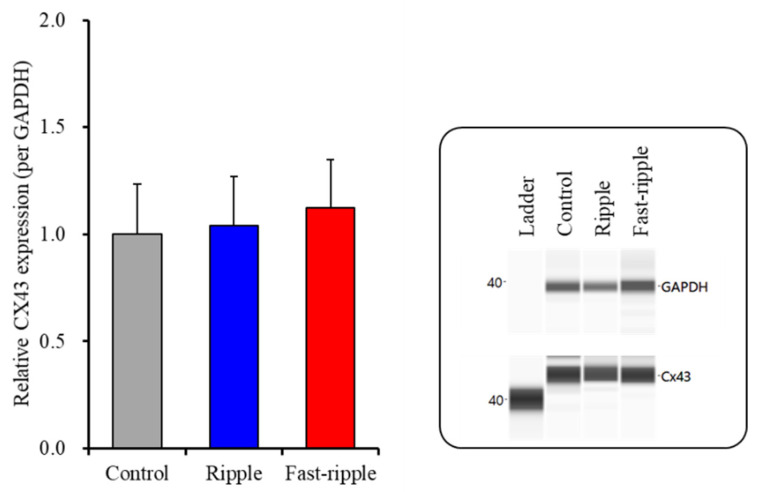
Effects of acute artificial ripple-HFO and fast-ripple-HFO evoked stimulations (100 sets) on the expression of Cx43 protein in the astroglial plasma membrane fraction. The right-side panel indicates the pseudo-gel images from the capillary immunoblotting results using anti-glyceraldehyde-3-phosphate dehydrogenase (GAPDH) and anti-connexin43 (Cx43) antibodies for blotting. In the left side panel, ordinate: mean ± SD (*n* = 6) of the relative protein level of Cx43 (per GAPDH).

After the 100 sets of ripple HFO or fast-ripple HFO-evoked stimulations, the proteins in the plasma membrane fractions of primary cultured astrocytes were extracted. The Cx43 protein expression in the astroglial plasma membrane fraction was not changed compared to the control (non-artificial HFO-evoked stimulation) (Figure 2). The expression of SV2A protein in the plasma membrane fraction could not be detected (data not shown).

These results of Study 1 suggest that astroglial Cx43-containing hemichannels do not release L-glutamate during the resting stage. Fast-ripple HFO acutely generates astroglial L-glutamate release via activation of Cx43-containing astroglial hemichannels, but ripple HFO cannot activate astroglial Cx43-containing hemichannels. Furthermore, neither ripple HFO nor fast-ripple HFO affects protein expressions of Cx43 or SV2A in the plasma membrane. Therefore, fast-ripple HFO acutely enhances astroglial transmission via the activation of hemichannels without affecting the functional hemichannel level.

#### 2.1.3. Concentration-Dependent Effects of Acute Administration of Brivaracetam and Levetiracetam on Fast-Ripple HFO-Evoked Astroglial L-Glutamate Release (Study 2)

It has been considered that the candidate therapeutic-relevant concentrations of brivaracetam and levetiracetam range from 2 to 6 μM and from 70 to 270 μM, respectively [35,36]. Based on these clinical findings, in the present study, to determine the concentration-dependent effects of brivaracetam and levetiracetam on astroglial L-glutamate release induced by 100 sets of fast-ripple-evoked stimulation, the cultured astrocytes were administered 3 μM (therapeutic-relevant range) and 10 μM (supratherapeutic range) brivaracetam, or 100 μM (therapeutic-relevant range) and 300 μM (supratherapeutic range) levetiracetam. The acute effects of brivaracetam and levetiracetam on astroglial transmission required exposure to these agents for longer than 120 min [13,34,37]. Based on these previous pre-clinical findings, in the present study, to determine the acute effects of brivaracetam and levetiracetam on fast-ripple HFO-evoked astroglial L-glutamate release, cultured astrocytes were administered brivaracetam and levetiracetam for 120 min prior to artificial HFO-evoked stimulation.

**Figure 3:**
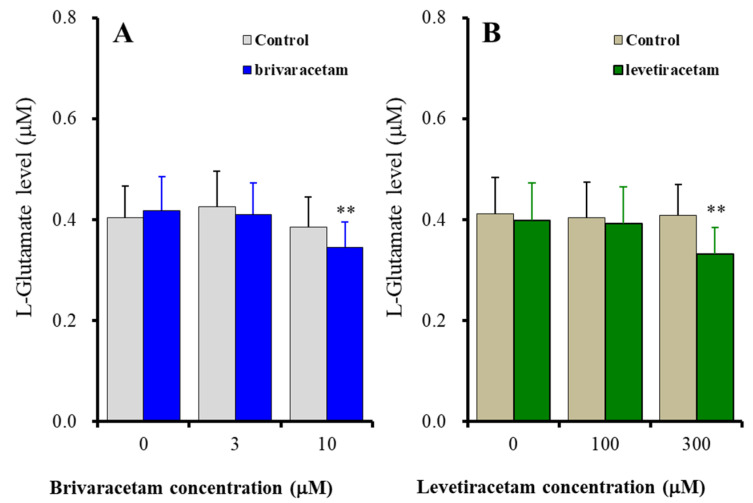
Concentration-dependent effects of acute administration (for 120 min) of brivaracetam (**A**) and levetiracetam (**B**) on astroglial L-glutamate release induced by 100 sets fast-ripple-HFO evoked stimulation. Ordinates indicate the mean ± SD of extracellular L-glutamate level (μM) (*n* = 6). Abscissas indicate the concentration of brivaracetam (blue columns) or levetiracetam (green columns) (μM). ** *p* < 0.01; relative to the brivaracetam or levetiracetam free (0) by MANOVA with Tukey’s post-hoc test.

The acute administration (for 120 min) of the therapeutic-relevant concentration of brivaracetam (3 μM) did not affect fast-ripple HFO-evoked astroglial L-glutamate release, whereas the supratherapeutic concentration (10 μM) suppressed fast-ripple HFO-evoked astroglial L-glutamate release [F_stimulation_(2, 20) = 23.21 (*p* < 0.01), F_brivaracetam_(1, 10) = 0.14 (*p* > 0.1), F_stimulation*brivaracetam_(2, 20) = 5.28 (*p* < 0.05)] (Figure 3A). Similar to brivaracetam, the acute administration of the therapeutic-relevant concentration of levetiracetam (100 μM) did not affect fast-ripple HFO-evoked astroglial L-glutamate release, whereas the supratherapeutic concentration (300 μM) suppressed fast-ripple HFO-evoked astroglial L-glutamate release [F_stimulation_(2, 20) = 12.91 (*p* < 0.01), F_levetiracetam_(1, 10) = 0.79 (*p* > 0.1), F_stimulation*levetiracetam_(2, 20) = 13.58 (*p* < 0.01)] (Figure 3B).

These results suggest that both brivaracetam and levetiracetam concentration-dependently suppress the activation of astroglial hemichannels induced by fast-ripple HFO. Supratherapeutic concentrations of brivaracetam and levetiracetam acutely inhibit the fast-ripple HFO-evoked activation of astroglial hemichannels, whereas therapeutic-relevant concentrations of these agents cannot affect astroglial hemichannel activation.

### 2.2. Subchronic Artificial HFO-Evoked Stimulations on Astroglial L-Glutamate Release

To clarify the subchronic effects of artificial HFO-evoked stimulation bursts on astroglial transmission, the cultured astrocytes were stimulated with 100 sets of artificial ripple HFO or fast-ripple HFO-evoked stimulations every 8 h for 1 day (100 *3 *1 sets during DIV27–28), 3 days (100 *3 *3 sets during DIV25–28), or 7 days (100 *3 *7 sets during DIV21–28).

#### 2.2.1. Subchronic Artificial HFO-Evoked Stimulations on Astroglial L-Glutamate Release through Activated Cx43-Containing Hemichannel (Study 3)

**Figure 4:**
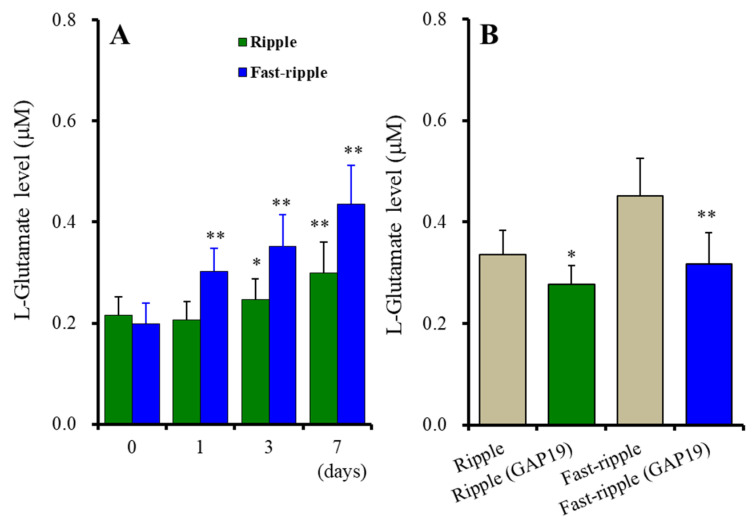
Effects of subchronic artificial ripple-HFO (green columns) and fast-ripple-HFO evoked (blue columns) stimulations for 1, 3, and 7 days on L-glutamate release from cultured astrocytes (**A**), and the inhibitory effects of TAT-GAP19 (green and blue columns) on subchronic artificial HFO-evoked astroglial L-glutamate release (**B**). Ordinates indicate the mean ± SD of extracellular L-glutamate level (μM) (*n* = 6). Abscissa in panel A indicates the days of HFO-evoked stimulations. In (**A**), * *p* < 0.05, ** *p* < 0.01; relative to the HFO-evoked stimulation free (0) by MANOVA with Tukey’s post-hoc test. In (**B**), * *p* < 0.05, ** *p* < 0.01; relative to ripple-HFO or fast-ripple-HFO evoked stimulation (control: TAT-GAP19 free) by Student’s *t*-test.

Contrary to acute stimulation, both chronic ripple HFO and fast-ripple HFO-evoked stimulations use-dependently enhanced astroglial L-glutamate release [F_stimulation_(1, 10) = 8.2 (*p* < 0.05), F_duration_(3, 30) = 127.9 (*p* < 0.01), F_stimulation*duration_(3, 30) = 30.8 (*p* < 0.01)] (Figure 4A). Chronic ripple HFO-evoked stimulation for 1 day did not affect astroglial L-glutamate release, but ripple HFO-evoked stimulation for longer than 3 days use-dependently increased astroglial L-glutamate release (Figure 4A). Similarly, fast-ripple HFO-evoked stimulation use-dependently increased astroglial L-glutamate release (ranged from 1 to 7 days) (Figure 4A). These subchronic ripple HFO and fast-ripple HFO-evoked stimulations enhanced astroglial L-glutamate release via the activation of astroglial Cx43-containing hemichannels, since both astroglial L-glutamate releases induced by subchronic ripple HFO and fast-ripple HFO-evoked stimulations (for 7days) were inhibited by 20 μM TAT-GAP19 (Figure 4B). The detailed experimental design is given in Section 4.3.3.

#### 2.2.2. Subchronic Administrations of Brivaracetam and Levetiracetam on Astroglial L-Glutamate Release Induced by Subchronic Artificial Ripple HFO-Evoked Stimulations (Study 4)

**Figure 5:**
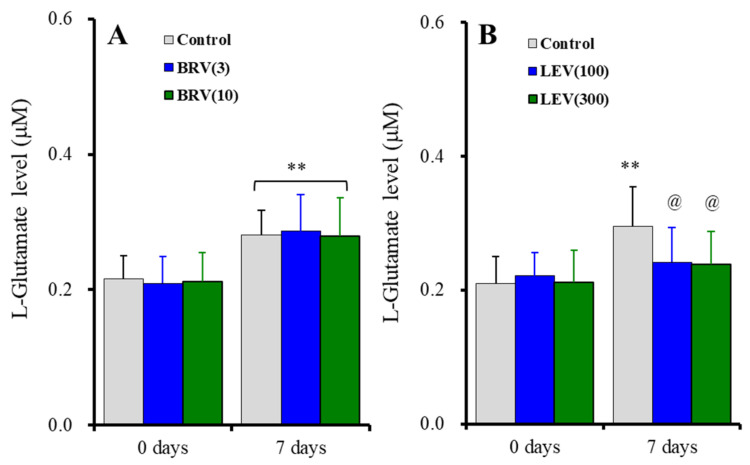
Concentration-dependent effects of subchronic administration of brivaracetam (0, 3, and 10 μM) (**A**) and levetiracetam (0, 100, and 300 μM) (**B**) for 7 days (DIV21-28) on astroglial L-glutamate release induced by subchronic ripple-HFO evoked stimulations (100 set) for 7 days. Ordinates indicate the mean ± SD of extracellular L-glutamate level (μM) (*n* = 6). ** *p* < 0.01; relative to the free of ripple-HFO evoked stimulation, and @ *p* < 0.05 relative to levetiracetam free (0) by MANOVA with Tukey’s post-hoc test.

The subchronic administration for 7 days (DIV21-28) of brivaracetam (3 and 10 μM) did not affect basal or subchronic ripple HFO-evoked astroglial L-glutamate release [F_duration_(1, 15) = 156.2 (*p* < 0.01), F_brivaracetam_(2, 15) = 0.1 (*p* > 0.1), F_duration*brivaracetam_(2, 15) = 0.4 (*p* > 0.1)] (Figure 5A). Contrary to brivaracetam, the subchronic administration for 7 days of levetiracetam (100 and 300 μM) did not affect basal astroglial L-glutamate release, but suppressed subchronic fast-ripple HFO-evoked (for 7 days) astroglial L-glutamate release [F_duration_(1, 15) = 71.3 (*p* < 0.01), F_levetiracetam_(2, 15) = 0.6 (*p* > 0.1), F_duration*levetiracetam_(2, 15) = 15.3 (*p* < 0.01)] (Figure 5B).

#### 2.2.3. Interaction between Subchronic Administrations of Brivaracetam and Levetiracetam and Ripple HFO-Evoked Stimulation on Protein Expression of Cx43 in the Plasma Membrane (Study 4)

**Figure 6:**
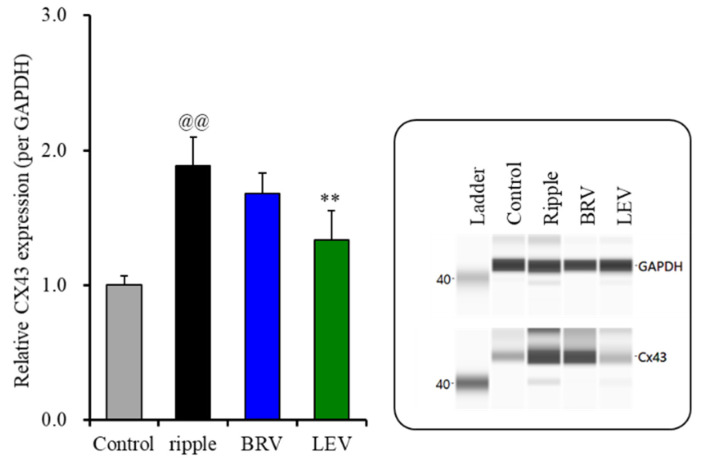
Effects of subchronic artificial ripple-evoked stimulations (100 sets for 7 days) on the expression of Cx43 protein in the astroglial plasma membrane fraction. The right-side panel indicates the pseudo-gel images from the capillary immunoblotting results using anti-GAPDH and anti-Cx43 antibodies for blotting. In the left side panel, ordinate: mean ± SD (*n* = 6) of the relative protein level of Cx43 (per GAPDH). @@ *p* < 0.01; relative to control, ** *p* < 0.01; relative to the ripple-HFO evoked stimulation by one-way ANOVA with Tukey’s post-hoc test.

The subchronic ripple HFO-evoked stimulations increased Cx43 protein expression in the astroglial plasma membrane (Figure 6). Contrarily, the protein expression of SV2A in the plasma membrane could not be detected during the resting stage or after the subchronic ripple HFO-evoked stimulation (data not shown). Subchronic administration of the therapeutic-relevant concentration of brivaracetam (3 μM) did not affect Cx43 protein expression, but the therapeutic-relevant concentration of levetiracetam (100 μM) decreased the increasing Cx43 protein expression in the astroglial plasma membrane fraction induced by ripple HFO-evoked stimulation [F(2, 15) = 35.9 (*p* < 0.01)] (Figure 6). Therefore, the therapeutic-relevant concentration of levetiracetam subchronically suppresses the ripple HFO-evoked astroglial L-glutamate release via the inhibition of increasing Cx43 expression in the plasma membrane, whereas that of brivaracetam has no affect.

#### 2.2.4. Subchronic Administrations of Brivaracetam and Levetiracetam on Astroglial L-Glutamate Release Induced by Subchronic Fast-Ripple HFO-Evoked Stimulations (Study 5)

**Figure 7:**
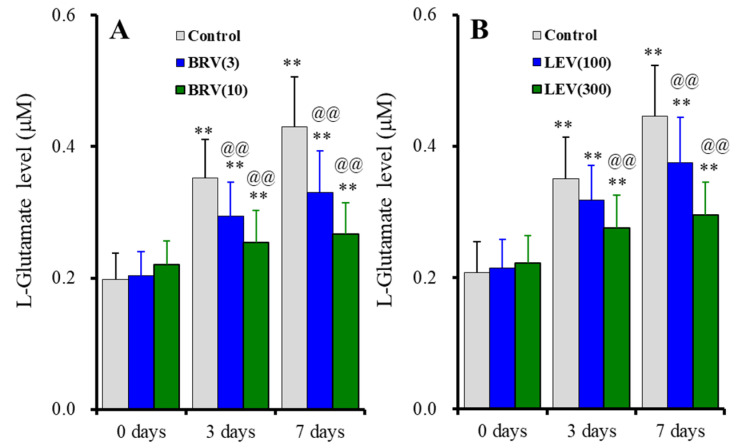
Concentration-dependent effects of subchronic administration of brivaracetam (0, 3 and 10 μM) (**A**) and levetiracetam (0, 100, and 300 μM) (**B**) for 7 days on astroglial L-glutamate release induced by subchronic fast-ripple-HFO evoked stimulations (100 set) for 3 or 7 days. Ordinates indicate the mean ± SD of extracellular L-glutamate level (μM) (*n* = 6). ** *p* < 0.01; relative to the free of fast-ripple-HFO evoked stimulation (0 days), and @@ *p* < 0.01; relative to free of brivaracetam or levetiracetam (control) by MANOVA with Tukey’s post-hoc test.

The subchronic administration (for 7 days) of brivaracetam (3 and 10 μM) suppressed astroglial L-glutamate release induced by subchronic fast-ripple HFO-evoked stimulations for 3 days and 7 days, without affecting basal release [F_duration_(2, 30) = 189.9 (*p* < 0.01), F_brivaracetam_(2, 15) = 4.0 (*p* < 0.05), F_duration*brivaracetam_(4, 30) = 29.5 (*p* < 0.01)] (Figure 7A). Similarly, subchronic administration of levetiracetam for 7 days (100 and 300 μM) also suppressed astroglial L-glutamate release induced by subchronic fast-ripple HFO-evoked stimulations for 3 days and 7 days, without affecting basal release [F_duration_(1.5, 21.9) = 328.6 (*p* < 0.01), F_levetiracetam_(2, 15) = 2.5 (*p* > 0.1), F_duration*levetiracetam_(2.9, 21.9) = 29.6 (*p* < 0.01)] (Figure 7B).

#### 2.2.5. Interaction between Subchronic Administrations of Brivaracetam and Levetiracetam and Fast-Ripple HFO-Evoked Stimulation on Protein Expression of Cx43 and SV2A in the Plasma Membrane (Study 5)

**Figure 8:**
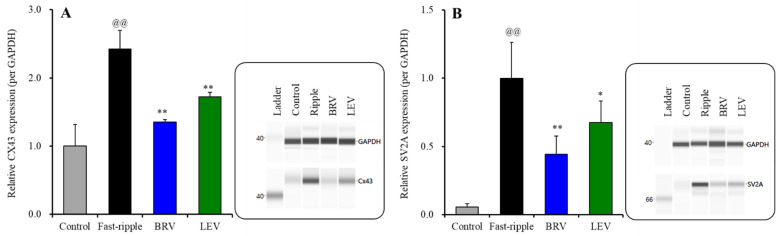
Interaction between subchronic artificial fast-ripple-HFO evoked stimulations (100 sets for 7 days) and therapeutic-relevant concentration of brivaracetam (3 μM) and levetiracetam (100 μM) on the protein expression of Cx43 (**A**) and synaptic vesicle protein 2A (SV2A) (**B**) in the astroglial plasma membrane fraction. The right-side panel indicates the pseudo-gel images from the capillary immunoblotting results using anti-GAPDH, anti-Cx43 and anti-SV2A antibodies for blotting. In the left side, ordinate: mean ± SD (*n* = 6) of the relative protein level of Cx43 and SV2A (per GAPDH). @@ *p* < 0.01; relative to control, * *p* < 0.05, ** *p* < 0.01; relative to the fast-ripple-HFO evoked stimulation (without agent) by one-way ANOVA with Tukey’s post-hoc test.

The subchronic fast-ripple HFO-evoked stimulation (for 7 days) increased protein expressions of Cx43 and SV2A in the plasma membrane of cultured astrocytes (Figure 8A,B). Expression of SV2A in the plasma membrane could not be detected during the resting stage (Figure 2) or after subchronic ripple HFO-evoked stimulation for 7 days (Figure 6), whereas subchronic fast-ripple HFO-evoked stimulation drastically increased expression of SV2A in the plasma membrane (Figure 8B).

The subchronic administration of the therapeutic-relevant concentration of brivaracetam (3 μM) inhibited the increasing expression of Cx43 [F(2, 15) = 56.7 (*p* < 0.01)] and SV2A [F(2, 15) = 46.1 (*p* < 0.01)] in the plasma membrane fraction induced by subchronic fast-ripple HFO-evoked stimulation (Figure 8A,B). Similar to brivaracetam, the subchronic administration of the therapeutic concentration of levetiracetam (100 μM) also inhibited the increasing expression of Cx43 [F(2, 15) = 51.4 (*p* < 0.01)] and SV2A [F(2, 15) = 43.6 (*p* < 0.01)] in the plasma membrane fraction by subchronic fast-ripple HFO-evoked stimulation (Figure 8A,B).

## 3. Discussion

### 3.1. Effects of Ripple HFO and Fast-Ripple HFO on Astroglial Transmission

Currently, HFOs are classified as ripple HFO (80–250 Hz) and fast-ripple HFO (250–500 Hz), depending on the electrophysiological frequency [27,38]. Functionally, the ripple HFO and fast-ripple HFO are considered to contribute to respective physiological sleep-related [31,39] and pathological epilepsy-related events [27,38], whereas the detailed acute/subchronic impacts of HFOs on transmission have not been clarified fully. The present study demonstrated the distinct effects between ripple HFO and fast-ripple HFO on astroglial L-glutamate release through Cx43-containing hemichannels in the astrocytes and Cx43 expression in the astroglial plasma membrane. Fast-ripple HFO acutely enhanced astroglial L-glutamate release through the hemichannel (hemichannel activation) without affecting the Cx43 expression in the astroglial plasma membrane, but subchronically enhanced both hemichannel activity and Cx43 expression in the plasma membrane. Ripple HFO also subchronically enhanced Cx43-containing hemichannel activity and Cx43 expression in the astroglial plasma membrane, but did not acutely affect hemichannel activity or Cx43 expression in the plasma membrane. These results indicate that fast-ripple HFO is more effective in astroglial hemichannel activation compared to ripple HFO, even if the amount of energy between ripple HFO and fast-ripple HFO is equal. Although the present study revealed that fast-ripple HFO enhanced astroglial L-glutamate release through the activated hemichannel, the mechanisms of increasing astroglial L-glutamate release induced by acute fast-ripple HFO-evoked stimulation have two possibilities; namely, the enhancement of hemichannel activity [13,24,25] and quantitative increasing functional hemichannel due to dissociation of gap junction [40]. To understand actual mechanisms of fast-ripple HFO-induced astroglial L-glutamate release, in the future, we shall determine the functions of connexin43-containing hemichannels and gap junctions before and after fast-ripple HFO-evoked stimulation, using their selective tracers.

Under physiological conditions, the expression and opening probability of hemichannels in normal tissues are low, whereas several pathological conditions drastically enhance expression and opening probability [18,19,22]. Increased expression of astroglial hemichannels in the regions close to epileptic focus of patients with epilepsy [41,42] and epileptic animal models [43,44,45,46] was observed. The opening probability of Cx43-containing astroglial hemichannels is strictly regulated by membrane potential and cation level imbalance between intracellular and extracellular spaces [19,24,25,26,44,47,48]. Indeed, the Cx43-containing hemichannel opens due to decreased extracellular Ca^2+^ lower than the 1 mM range during neuronal hyperactivity [13,26,44,49]. Increasing extracellular K^+^ level enhances Cx43-containing hemichannel activity [26,44]. Neuronal hyperactivity, such as epileptic discharge, generates cation level imbalance between extracellular and intracellular spaces, which activates Cx43-containing hemichannels [19,26,44]. During the opening of the Cx43-containing hemichannel, its opening probability is rapidly increased and decreased by depolarisation and hyperpolarisation, respectively [47]. Therefore, Cx43-containing hemichannels can be functioning as sensors for cation level and membrane potential in the signalling from neurons to astrocytes. Notably, the active state of astroglial hemichannels is persistent compered to neuronal voltage-sensitive and ligand-gated cation channels [13,19,24,26]. These findings suggest that the activation of astroglial hemichannels plays an important role in ictogenesis and epileptic neuronal damage, since an activated hemichannel releases large amounts of various excitatory modulators, such as L-glutamate, D-serine, ATP, K^+^, and eicosanoids [18,19,25].

Several studies support the implication of astroglial Cx43-containing hemichannels in ictogenesis and seizure-induced neuronal damage. Acute systemic administration of TAT-GAP19, which selectively inhibits the Cx43-containing hemichannel without affecting the Cx43-containing gap junction, and carbenoxolone, a non-selective inhibitor of both hemichannel and gap junction, suppress the duration and severity of several epileptic models [50,51]. In addition to these findings, we previously demonstrated that activation of astroglial Cx43-containing hemichannels played a fundamental role in the development of not only ictogenesis but also epileptogenesis in carbamazepine-resistant ADSHE model rats [19,26,44,52,53]. Before ADSHE onset, the combination of neuronal excitability induced by GABAergic disinhibition and physiological ripple HFO lead to the upregulation and activation of astroglial hemichannels [26]. These functional abnormalities have been already observed in the embryonic brain of ADSHE rat models, but these pathological conditions were reversible, since suppression of neuronal hyperactivation improved these astroglial hyperactivations [26]. ADSHE onset needs to produce interictal discharges including fast-ripple HFO during the critical period of ADSHE onset [19,44,52]. Furthermore, after ADSHE onset, both physiological sleep-related ripple HFO and epileptogenic fast-ripple HFO can contribute to ictogenesis or the generation of ADSHE seizures; however, the contribution of fast-ripple HFO to ADSHE seizure development was much greater than that of ripple HFO [19,26,44,52,53]. Therefore, the fast-ripple HFO is probably directly involved in the pathomechanisms of epileptogenesis and ictogenesis, whereas the ripple HFO is principally involved in physiological electric events, but it can also contribute to the development of epileptogenesis and/or ictogenesis in the presence of primary pathogenesis of epilepsy. Taken together with the pathomechanisms of genetic ADSHE model rats, the results of this study suggest that the HFOs probably play important roles in the developments of epileptogenesis and ictogenesis via pathological activations of astroglial hemichannels.

### 3.2. Effects of Brivaracetam and Levetiracetam on Fast-Ripple HFO-Induced Changing Astroglial Functions

The present study identified similar effects of brivaracetam and levetiracetam on fast-ripple HFO-evoked activation of astroglial hemichannel functions. Subchronic administration of brivaracetam and levetiracetam inhibited the subchronic fast-ripple HFO-evoked activation of Cx43-containing hemichannels and increasing Cx43 expression in the plasma membrane. Expression of Cx43 in the plasma membrane is regulated by the trafficking processes via several intracellular signalling pathways, such as Erk, Akt, and adenosine monophosphate-activated protein kinase (AMPK) [23,33,54,55]. Brivaracetam suppressed Cx43 trafficking to the plasma membrane [13] and persistent depolarisation activated Cx43 trafficking to the plasma membrane, which is predominantly regulated by Erk signalling [26,44]. Levetiracetam prevented brain damage via the suppression of Erk signalling [56]. Therefore, the inhibitory effects of subchronic administration of brivaracetam and levetiracetam on Cx43 trafficking to the plasma membrane are probably modulated by inhibition of Erk signalling. To clarify the detailed mechanisms of inhibition of Cx43 trafficking via Erk signalling inhibition by brivaracetam and levetiracetam, further studies are needed.

Another possibility regarding the mechanism of increasing Cx43 expression in the plasma membrane has been reported. SV2A selectively expresses in the vesicular membrane, and contributes to transmitter release as a Ca^2+^ sensor in the Ca^2+^-dependent exocytosis mechanisms [14]; however, the hyperactivation of fusion/docking of the plasma membrane with vesicular membrane through the exocytosis process produces the ectopic SV2A in the plasma membrane [13,16]. Indeed, in the present study, the subchronic fast-ripple HFO increased ectopic SV2A in the plasma membrane fraction. The luminal domain of ectopic SV2A becomes stabilising in the plasma membrane due to interaction with the extracellular matrix [16] or plasma membrane protein [13]. Cx43 is also a candidate target protein of ectopic SV2A [13]. In particular, the increasing ectopic SV2A in the astroglial plasma membrane is associated with the prolongation of the half-life of Cx43 in the astroglial plasma membrane [13]. Turnover of Cx43 is faster than other cation channel proteins, and the half-life is several hours [18]. The initiation process in the Cx43 degradation is internalisation [18]. Therefore, the extracellular matrix between ectopic SV2A and Cx43 probably enhances astroglial transmitter release by increasing the total amount of activated hemichannel levels induced by the prolongation of turnover of Cx43 in the plasma membrane. Based on the findings, the inhibition of astroglial exocytosis via the suppression of SV2A function and subsequent secondary inhibition of turnover prolongation of activated hemichannels via the suppression of increasing ectopic SV2A in the plasma membrane are probably involved in the antiseizure mechanisms of action of brivaracetam and levetiracetam.

Although acute fast-ripple HFO-evoked stimulation enhanced activated hemichannel permeability without increasing expression of Cx43 or SV2A in the plasma membrane, the acute administration of the supratherapeutic concentration of brivaracetam and levetiracetam unexpectedly suppressed the fast-ripple HFO-evoked astroglial L-glutamate release. These results suggest that both brivaracetam and levetiracetam directly inhibit depolarisation-induced activation of hemichannel activity. This direct inhibition of Cx43-containing hemichannel activity induced by brivaracetam and levetiracetam is interesting for the interpretation of novel antiseizure strategies, whereas these inhibitory effects of brivaracetam and levetiracetam on acute fast-ripple HFO-induced activation of hemichannels probably do not contribute to their antiseizure actions, since the therapeutic-relevant concentration could not supress acute fast-ripple HFO-evoked astroglial L-glutamate release. Unfortunately, the present study cannot explain the detailed mechanisms of inhibitory effects of brivaracetam and levetiracetam on acute fast-ripple HFO responses. We shall explore these mechanisms in detail in the future.

Extracellular alkaline and acidic shift enhances and supresses Cx43-containing hemichannel activity, respectively [26]. Indeed, zonisamide supresses carbamazepine-resistant epileptic seizures by extracellular acidification via carbonic anhydrase inhibition [26,57,58]. SV2 is considered to be the mainstay of luminal protein for the matrix with other vesicular proteins in the synaptic vesicle membrane due to the length of its luminal domain, which is longer than the diameter of the vesicle lumen [59]. Intracellular acidic shift promotes adsorption among SV2A and other vesicular proteins via negatively charged proteoglycans [60]. Although the impacts of extracellular acidic shift on extracellular matrix formation of ectopic SV2A in the plasma membrane has not been clarified, based on the evidence, the intracellular acidic shift promotes the interaction of SV2A with vesicular proteins. It is easily speculated that extracellular acidic shift also enhances adsorption to the extracellular protein matrix. Therefore, the extracellular acid shift probably affects contradictorily activated tripartite synaptic transmission induced by HFO, since extracellular acidic shift suppresses Cx43-containing hemichannel activity, but expands the half-life of activated Cx43-containing hemichannel on the astroglial plasma membrane via enhancement of the extracellular protein matrix absorption between Cx43 and SV2A. The above hypothesis possibly provides a novel strategy as a rational combination therapy, adding-on SV2A inhibitor to carbonic anhydrase inhibiting antiseizure drugs. We shall report regarding the interaction between SV2A inhibitor and carbonic anhydrase inhibitor on tripartite synaptic transmission in further study.

### 3.3. Candidate Mechanisms of Adverse Behavioural Effects of Brivaracetam and Levetiracetam Associated with Astroglial Hemichannel

In contrast to fast-ripple HFO, the ripple HFO is usually observed after the sensorimotor/cognitive tasks and during physiological sleep-related electrophysiological events (sleep spindle burst) [38]. Recent psychophysiological studies have accumulated findings that ripple HFO and sleep spindle play important roles in the generation of several subdomains of cognition, such as perceptual/sensory integration, reasoning ability, and memory consolidation [29,61,62]. Therefore, antiseizure drug medications that selectively inhibit fast-ripple HFO without affecting ripple HFO represent an essential strategy for improving the quality of life of patients with epilepsy. Several antiseizure drugs have been associated with adverse cognitive reactions—related to attention, memory, learning, and language processing—in a dose-dependent manner [63,64,65]. A recent preclinical study revealed that carbamazepine concentration-dependently suppressed ripple HFO [66]. Taken together with clinical findings, the inhibition of ripple HFO and fast-ripple HFO probably underlies the antiseizure actions and cognitive impairments observed with therapeutic and supratherapeutic doses of antiseizure drugs.

The effects of brivaracetam on ripple HFO and fast-ripple HFO remain to be clarified clinically and preclinically. The effects of levetiracetam on ripple HFO or sleep spindle remain to be clinically clarified; however, two preclinical studies revealed that levetiracetam predominantly inhibited fast-ripple HFO compared to ripple HFO [30,31]. These electrophysiological profiles of levetiracetam demonstrated by two preclinical physiological studies suggest that levetiracetam is a possible candidate antiseizure drug with reasonable pharmacological properties, predominantly suppressing fast-ripple HFO compared to ripple HFO. Indeed, both brivaracetam and levetiracetam clinically displayed the best antiepileptic efficacies and safety profiles in the class of antiseizure drugs [3,4,5]; however, these two agents have been listed as high-risk antiseizure drugs regarding aggressive behaviour [6,7,8,9,10]. In the present study, we identified the mutual antiseizure mechanisms between brivaracetam and levetiracetam: both agents inhibit the synergistic interaction between ectopic SV2A and activated astroglial hemichannels induced by fast-ripple HFO, whereas the effects of brivaracetam and levetiracetam on the astroglial responses to ripple HFO were not identical.

Subchronic administration of therapeutic-relevant concentrations of levetiracetam prevented the enhancement of astroglial L-glutamate release through activated Cx43-containing hemichannels induced by subchronic ripple HFO exposure, whereas therapeutic-relevant concentrations of brivaracetam had no effect. This discrepant effect between brivaracetam and levetiracetam on the activation of astroglial Cx43-containing hemichannels induced by subchronic ripple HFO suggests a candidate mechanism of the cognitive impairment induced by levetiracetam. Recent psychopharmacological studies indicate the possibility that tripartite synaptic transmission, including astroglial transmission release through hemichannels, plays an important role in the regulation of cognition and emotional functions [19,22,23,33,54,55].

Furthermore, five clinical studies reported that more than 65% of patients who experienced adverse behavioural/psychiatric effects induced by levetiracetam improved their cognitive dysfunctions (irritability, aggression, depression) by switching to brivaracetam [32,67,68,69,70,71]. Therefore, in order to understand the detailed mechanism of behavioural/psychological adverse effects of antiseizure drugs, in addition to the analysis of the effect of antiseizure drugs on the expression of ripple HFO, the determination of the effects of astroglial transmission responses associated with hemichannel to physiological ripple HFO possibly provides novel findings regarding the pathophysiology of antiseizure drug-induced behavioural/psychological adverse effects. Levetiracetam and brivaracetam are members of the racetam class of antiepileptic drugs that selectively bind and probably inhibit SV2A [1,14]. The major antiseizure mechanisms of brivaracetam and levetiracetam have been considered to bind SV2A, but the pharmacological profiles of these agents are not identical [13,14]. The binding affinity of brivaracetam to SV2A is about 30 times higher than that of levetiracetam [1,14]. Furthermore, levetiracetam weakly inhibits voltage-dependent K^+^ and Ca^2+^ channels, whereas brivaracetam inhibits Na^+^ currents through voltage-dependent sodium channels [1,14,34]. In addition to the differences in pharmacological profiles against cation channels, the different effects between levetiracetam and brivaracetam on activated astroglial hemichannel induced by ripple HFO might explain the differences in adverse behavioural/psychiatric reactions of these two agents.

## 4. Materials and Methods

### 4.1. Chemical Agents

The selective Cx43 inhibitor, N-terminal transactivator of transcription GAP19 (TAT-GAP19) [50] was obtained from Funakoshi (Tokyo, Japan). Brivaracetam and levetiracetam were obtained from Cosmo Bio (Tokyo, Japan) and FUJIFILM Wako Chemicals (Osaka, Japan), respectively. All agents used in this study were dissolved in medium directly.

### 4.2. Preparation of Primary Cultured Astrocytes

All animal care and experimental procedures described in this report complied with the Ethical Guidelines established by the Institutional Animal Care and Use Committee at Mie University, Japan (No. 29–22-R3, 31 October 2019) and are reported in accordance with the Animal Research: Reporting of In Vivo Experiments (ARRIVE) guidelines. Astrocytes were prepared using a protocol adapted from previously described methods.

Pregnant Sprague-Dawley rats (*n* = 30) were housed individually in cages, in air-conditioned rooms (temperature, 22 ± 2 °C) with a 12 h/12 h light/dark cycle, and provided access to food and water ad libitum. To prepare cortical astrocyte cultures, the neonatal rats were sacrificed by decapitation at 0–24 h of age. The cerebral hemispheres of the animals were removed under a dissecting microscope, following which the tissue was chopped into fine pieces using scissors and triturated briefly with a micropipette. The suspension was filtered using a 70 µm nylon mesh (BD Biosciences, Franklin Lakes, NJ, USA) and centrifuged. The pellets obtained were resuspended in 10 mL Dulbecco’s modified Eagle’s medium (Sigma-Aldrich, St. Louis, MO, USA) containing 10% foetal calf serum (fDMEM). At DIV14, the contaminating cells were removed by shaking in a standard incubator for 16 h at 200 rpm. On DIV21, the astrocytes were removed from the flasks by means of trypsinisation and seeded onto translucent PET membrane (1.0 µm) placed directly in 24-well plates (BD Biosciences) at a density of 5 × 105 cells/cm^2^ for experiments. To subchronically administer brivaracetam and levetiracetam, these agents were added to fDMEM. At DIV28, the cultured astrocytes were washed out using artificial cerebrospinal fluid (ACSF: 130 mM NaCl, 5.4 mM KCl, 1.8 mM CaCl_2_, 1 mM MgCl_2_, and 5.5 mM glucose, buffered with 20 mM HEPES buffer to pH 7.3). After the wash-out, the astrocytes were incubated in ACSF (100 μL on a translucent PET membrane) at 35 °C for 60 min in a CO_2_ incubator (pre-incubation).

### 4.3. Artificial HFO-Evoked Stimulation and Study Designs

Both ripple-HFO and fast-ripple-HFO evoked stimulations were performed using a busdrive amplifier (SEG-3104MG, Miyuki Giken, Tokyo, Japan). Both ripple-evoked and fast-ripple-evoked stimulations were set at a square-wave DC pulse output, with a magnitude of 300 mV/mm^2^ [26,72]. Ripple-HFO evoked stimulation was composed of 10 stimuli at 200 Hz and 10 bursts (50% duty cycle) at burst intervals of 100 ms per 1 s. Fast-ripple-HFO evoked stimulation was composed of 10 stimuli at 500 Hz and 10 bursts (50% duty cycle) at burst intervals of 40 ms per 1 s. These ripple-HFO and fast-ripple-HFO evoked stimulations were regulated using LabChart version 8.2 software (AD Instruments, Dunedin, New Zealand). The energisation amounts during the ripple-HFO and fast-ripple-HFO evoked stimulations were set to be equal.

#### 4.3.1. Acute Effects of Artificial HFO-Evoked Stimulation on Astroglial L-Glutamate Release (Study 1)

To clarify the use-dependent effects of acute HFO-evoked stimulations on astroglial L-glutamate release, at DIV28, the cultured astrocytes were stimulated with ripple-HFO or fast-ripple-HFO evoked stimulations (10, 30, or 100 sets) in ACSF. After 20 min of HFO-evoked stimulations, the ACSF was collected for determination of L-glutamate release. To specifically identify the use-dependent effects of HFO on astroglial L-glutamate release through activated Cx43 containing hemichannels, during ripple-HFO and fast-ripple-HFO evoked stimulations, the cultured astrocytes were incubated in ACSF, with or without (control) 20 μM TAT-GAP19 (selective connexin43 inhibitor). After the collection of ACSF, the plasma membrane fraction of the cultured astrocytes was extracted.

#### 4.3.2. Concentration-Dependent Effects of Acute Administration of Brivaracetam and Levetiracetam on Artificial Fast-Ripple-HFO Evoked Stimulations on Astroglial L-glutamate Release (Study 2)

To clarify the acute administration of brivaracetam and levetiracetam on 100 sets, fast-ripple-HFO evoked astroglial L-glutamate release; at DIV28, the cultured astrocytes were incubated in the ACSF including brivaracetam (0, 3 or 10 μM) or levetiracetam (0, 100 or 300 μM) for 120 min. After incubation, cultured astrocyte was stimulated by fast-ripple-HFO for 100 sets.

#### 4.3.3. Subchronic Artificial HFO-Evoked Stimulations on Astroglial L-Glutamate Release (Study 3)

To clarify the effects of subchronic HFO-bursts on astroglial L-glutamate release, during DIV21–28, the cultured astrocytes were stimulated with 100 sets of artificial ripple-HFO or fast-ripple-HFO evoked stimulations for 8 h for 1 day (100*3*1 sets during DIV27–28), 3 days (100 *3 *3 sets during DIV25–28), or 7 days (100 *3 *7 sets during DIV21–28). At DIV28, after the pre-incubation period, the cultured astrocytes were stimulated with ripple-HFO or fast-ripple-HFO evoked stimulations (100 sets) in ACSF. To specifically identify the subchronic use-dependent effects of artificial HFO-evoked stimulations on astroglial L-glutamate release through activated Cx43-containing hemichannels, during ripple-HFO and fast-ripple-HFO evoked stimulations, the cultured astrocytes were incubated in ACSF, with or without (control) 20 μM TAT-GAP19 [26]. After 20 min of HFO-burst stimulations, the ACSF was collected for determination of L-glutamate release.

#### 4.3.4. Subchronic Administrations of Brivaracetam and Levetiracetam on Astroglial L-Glutamate Release Induced by Subchronic Artificial Ripple-HFO Evoked Stimulation (Study 4)

To clarify the effects of subchronic administration of brivaracetam and levetiracetam on subchronic ripple-HFO evoked stimulation on astroglial L-glutamate release, during DIV21–28, the cultured astrocytes were incubated in the ACSF containing with brivaracetam (0, 1, or 3 μM) or levetiracetam (0, 100, or 300 μM), and stimulated with 100 sets of artificial ripple-HFO evoked stimulations for 8 h for 1 day (100*3*1 sets during DIV27–28), 3 days (100 *3 *3 sets during DIV25–28), or 7 days (100 *3 *7 sets during DIV21–28). At DIV28, the cultured astrocytes were stimulated with ripple-HFO evoked stimulations (100 sets) in ACSF. After the collection of ACSF, the plasma membrane fraction of the cultured astrocytes was also extracted.

#### 4.3.5. Subchronic Administrations of Brivaracetam and Levetiracetam on Astroglial L-Glutamate Release Induced by Subchronic Artificial Fast-Ripple-HFO Evoked Stimulation (Study 5)

To clarify the effects of subchronic administration of brivaracetam and levetiracetam on subchronic fast-ripple-HFO evoked stimulation on astroglial L-glutamate release, during DIV21–28, the cultured astrocytes were incubated in the ACSF containing with brivaracetam (0, 1, or 3 μM) or levetiracetam (0, 100, or 300 μM), and stimulated with 100 sets of artificial fast-ripple-HFO evoked stimulations for 8 h for 1 day (100*3*1 sets during DIV27–28), 3 days (100 *3 *3 sets during DIV25–28), or 7 days (100 *3 *7 sets during DIV21–28). At DIV28, the cultured astrocytes were stimulated with fast-ripple-HFO evoked stimulations (100 sets) in ACSF. After the collection of ACSF, the plasma membrane fraction of the cultured astrocytes was also extracted.

### 4.4. Determination of L-Glutamate Levels

L-glutamate levels were determined using ultra-high-performance liquid chromatography (xLC3185PU, Jasco, Tokyo, Japan) with fluorescence resonance energy transfer detection (xLC3120FP, Jasco), after dual derivatisation with isobutyryl-L-cysteine and o-phthalaldehyde. Derivative reagent solutions were prepared by dissolving isobutyryl-L-cysteine (2 mg) and o-phthalaldehyde (2 mg) in 0.1 mL ethanol, followed by the addition of 0.9 mL sodium borate buffer (0.2 M, pH 9.0). Automated pre-column derivatives were prepared by drawing up a 5 μL aliquot of the sample, standard, or blank solution, and 5 μL of derivative reagent solution, and allowing the two to react in reaction vials for 5 min before injection. The derivatised samples (5 μL) were injected using an autosampler (xLC3059AS, Jasco). The analytical column (Triat C18, particle 1.8 µm, 50 mm × 2.1 mm, YMC, Kyoto, Japan) was maintained at 45 °C, with the flow rate set at 500 μL/min. A linear gradient elution program was performed over 10 min with mobile phases A (0.05 M citrate buffer, pH 5.0) and B (0.05 M citrate buffer containing 30% acetonitrile and 30% methanol, pH 3.5). The excitation/emission wavelengths of the fluorescence detector were set at 345/455 nm. To correct the deviations of L-glutamate level due to the cultured cell number, after the experiments, the total protein level was determined using Protein Assay Reagent kit (FUJIFILM Wako Pure Chemical Corporation; Osaka, Japan).

### 4.5. Capillary Immunoblotting

To study the effects of HFO-evoked stimulation on the expression of Cx43 and SV2A in the plasma membrane fraction, the cultured astrocytes were extracted using a Minute Plasma Membrane Protein Isolation Kit (Invent Biotechnologies, Plymouth, MN, USA). Capillary immunoblotting analysis was performed using Wes (ProteinSimple, Santa Clara, CA, USA), according to the ProteinSimple user manual. The plasma membrane fraction of primary cultured astrocytes was mixed with a master mix (ProteinSimple) until a final concentration of 1 × sample buffer, 1 × fluorescent molecular weight marker, and 40 mM dithiothreitol was obtained, and then heated at 95 °C for 5 min. The samples, blocking reagent, primary antibodies, horseradish peroxidase (HRP)-conjugated secondary antibody, chemiluminescent substrate (SuperSignal™ West Femto; Thermo Fisher Scientific, Waltham, MA, USA); additionally, separation and stacking matrices were also dispensed into designated wells in a 25-well plate. After plate loading, separation electrophoresis and immunodetection steps were performed in the capillary system, which was fully automated. Capillary immunoblotting analysis was performed at room temperature with the default settings of the instrument. Capillaries were first filled with a separation matrix followed by a stacking matrix and approximately 40 nL sample loading. During electrophoresis, the proteins were separated on the basis of molecular weight through the stacking and separation matrices, at 250 V for 40 min, and then immobilised on the capillary wall using proprietary photo-activated capture chemistry. The matrices were then washed. Following that, the capillaries were incubated with a blocking reagent for 15 min, and target proteins were probed with primary antibodies, followed by incubation with HRP-conjugated secondary antibodies (anti-rabbit HRP-conjugated IgG, A00098, 10 μg/mL, GenScript, Piscataway, NJ, USA). Antibodies against GAPDH (NB300–322, 1:100, Novus Biologicals, Littleton, CO, USA), connexin43 (C6219, 1:100, Sigma-Aldrich), Erk (AF1576, 10 μg/mL, R&D Systems, Minneapolis, MN, USA), and SV2A (ab32942, 1: 500, Abcam, Cambridge, UK) were diluted in Immuno Shot Platinum (CosmoBio, Tokyo, Japan).

### 4.6. Data Analysis

All experiments were designed such that the groups were equal size (*n* = 6), without carrying out formal power analysis, according to previous studies [13,26,34]. All values are expressed as mean ± SD. A two-tailed *p*-value of less than <0.05 was considered statistically significant. Drug concentrations of acute and subchronic administrations were also selected according to a previous study [13,24,26,34]. Where possible, we sought to randomise and blind the sample data. In particular, to determine the levels of L-glutamate and protein expression, the sample order was set on the autosampler, according to a random number table.

The levels of astroglial L-glutamate release and protein expression levels were analysed using one-way analysis of variance (ANOVA), multivariate analysis of variance (MANOVA) or Student’s *t*-test with BellCurve for Excel version 3.2 (Social Survey Research Information Co., Ltd., Tokyo, Japan). The F-value of MANOVA was analysed using sphericity-assumed degrees of freedom. When the F-value of ANOVA or MANOVA were significant, the data were analysed using Tukey’s post-hoc test with BellCurve for Excel.

## 5. Conclusions

In the present study, we explored the effects of brivaracetam and levetiracetam on pathological fast-ripple HFO- and physiological ripple HFO-induced astroglial transmission to clarify the mechanisms of antiseizure and adverse behavioural/psychiatric effects of brivaracetam and levetiracetam. Acute ripple HFO did not affect Cx43 expression in the plasma membrane or astroglial hemichannel activity; however, subchronic ripple HFO-evoked stimulation enhanced astroglial hemichannel activity and Cx43 expression in the plasma membrane. In contrast to ripple HFO, the fast-ripple HFO acutely activated the astroglial Cx43-containing hemichannel without affecting functional Cx43 expression in the plasma membrane, whereas subchronic fast-ripple HFO-evoked stimulation activated astroglial hemichannel and increased Cx43 expression in the plasma membrane. Furthermore, subchronic fast-ripple HFO produced ectopic SV2A expression in the plasma membrane. Subchronic administrations of brivaracetam and levetiracetam inhibited fast-ripple HFO-evoked astroglial L-glutamate release and Cx43 expression in the plasma membrane. Subchronic administrations of levetiracetam also inhibited ripple HFO-evoked astroglial L-glutamate release and Cx43 expression in the plasma membrane, whereas subchronic administrations of brivaracetam did not affect ripple HFO-evoked astroglial L-glutamate release and Cx43 expression. These results suggest that the inhibitory effects of both therapeutic-relevant concentrations of brivaracetam and levetiracetam on fast-ripple HFO-induced astroglial responses are at least partially involved in the mutual mechanisms of antiseizure actions of brivaracetam and levetiracetam. Contrarily, the discrepant effects between brivaracetam and levetiracetam on ripple HFO-induced astroglial responses suggest that the inhibition of ripple HFO astroglial responses probably contributes to the adverse behavioural/psychiatric effects of levetiracetam.

## Data Availability

The data that support the findings of this study are available from the corresponding author upon reasonable request. Some data may not be made available because of ethical restrictions.
